# Effects of SGLT2 inhibitors on cardiac function and health status in chronic heart failure: a systematic review and meta-analysis

**DOI:** 10.1186/s12933-023-02042-9

**Published:** 2024-01-03

**Authors:** Jiao Chen, Chunxia Jiang, Man Guo, Yan Zeng, Zongzhe Jiang, Dongmin Zhang, Mengqin Tu, Xiaozhen Tan, Pijun Yan, XunMei Xu, Yang Long, Yong Xu

**Affiliations:** 1 Department of Endocrinology and Metabolism, the Affiliated Hospital of Southwest Medical University, Luzhou, Sichuan China; 2 Department of Endocrinology, The Third Hospital of Mianyang, Sichuan Mental Health Center, Mianyang, Sichuan China; 3 Sichuan Clinical Research Center for Nephropathy, Luzhou, Sichuan China; 4 Metabolic Vascular Disease Key Laboratory of Sichuan Province, Luzhou, China; 5 State Key Laboratory of Quality Research in Chinese Medicine, Macau University of Science and Technology, Macao, China; 6 Faculty of Chinese Medicine, Macau University of Science and Technology, Macao, China

**Keywords:** SGLT2 inhibitors, Cardiac function, Health status, Chronic Heart Failure, Meta-analysis

## Abstract

**Purpose:**

Numerous clinical studies have explored sodium–glucose cotransporter 2 inhibitor (SGLT2i) in patients with chronic heart failure (CHF), with or without type 2 diabetes mellitus (T2DM), and SGLT2i were proved to significantly reduce CHF hospitalization, cardiovascular death, cardiovascular mortality, all-cause mortality and myocardial infarction in patients with or without T2DM. However, only a limited few have investigated the effects of SGLT-2i on HF disease-specific health status and cardiac function. This meta-analysis aims to assess the effects of SGLT2i on disease-specific health status and cardiac function in CHF patients.

**Methods:**

A comprehensive search was conducted of trials by searching in PubMed, EMBASE, CENTRAL, Scopus, and Web of Science, and two Chinese databases (CNKI and Wanfang), Clinical Trials (http://www.clinicaltrials.gov) were also searched.

**Results:**

A total of 18 randomized controlled trials (RCTs) involving 23,953 participants were included in the meta-analysis. The effects of SGLT2 inhibitors were compared with control or placebo groups in CHF with or without T2DM. The SGLT2 inhibitors group exhibited a significant reduction in pro b-type natriuretic peptide (NT-proBNP) levels by 136.03 pg/ml (95% confidence interval [CI]: −253.36, − 18.70; P = 0.02). Additionally, a greater proportion of patients in the SGLT2 inhibitors group showed a ≥ 20% decrease in NT-proBNP (RR = 1.45, 95% CI [0.92, 2.29], p = 0.072). However, no statistically significant difference was observed for the effects on B-type natriuretic peptide (BNP). The use of SGLT-2 inhibitors led to a noteworthy improvement in LVEF by 2.79% (95% CI [0.18, 5.39];P = 0.036). In terms of health status, as assessed by the Kansas City Cardiomyopathy Questionnaire (KCCQ) and 6-minute walk distance, SGLT2 inhibitors led to a significant improvement in KCCQ clinical summary (KCCQ-CS) score (WMD = 1.7, 95% CI [1.67, 1.73], P < 0.00001), KCCQ overall summary (KCCQ-OS) score (WMD = 1.73, 95% CI [0.94, 2.52], P < 0.00001), and KCCQ total symptom (KCCQ-TS) score (WMD = 2.88, 95% CI [1.7, 4.06], P < 0.00001). Furthermore, the occurrence of KCCQ-CS and KCCQ-OS score increases ≥ 5 points had relative risks (RR) of 1.25 (95% CI [1.11, 1.42], P < 0.00001) and 1.15 (95% CI [1.09, 1.22], P < 0.00001), respectively. Overall, SGLT2 inhibitors increased the 6-minute walk distance by 23.98 m (95% CI [8.34, 39.62]; P = 0.003) compared to control/placebo from baseline.

**Conclusions:**

The SGLT2 inhibitors treatment offers an effective strategy for improving NT-proBNP levels, Kansas City Cardiomyopathy Questionnaire scores and 6-minute walk distance in CHF with or without T2DM. These findings indicate that SGLT2i improve cardiac function and health status in CHF with or without T2DM, and provide valuable guidance for clinicians making treatment decisions for patients with CHF.

**Supplementary Information:**

The online version contains supplementary material available at 10.1186/s12933-023-02042-9.

## Introduction

Chronic heart failure (CHF) is a terminal state of various heart diseases, with high morbidity, hospitalization rate and fatality rate. Based on the left ventricular ejection fraction (LVEF), CHF can be categorized into heart failure with reduced ejection fraction (HFrEF, LVEF < 40%), heart failure with preserved ejection fraction (HFpEF, LVEF ≥ 50%), and heart failure with mildly reduced ejection fraction (HFmrEF,40% ≤ LVEF < 50%) [1]. In the United States and Western Europe, heart failure remains the leading cause of hospitalization [2]. Drug therapy is an important and critical measure to improve the quality of life and prolong the survival of patients with CHF. The conventional medical treatment for CHF, often referred to as the “golden triangle”, includes angiotensin-converting enzyme inhibitors (ACEIs)/angiotensin II receptor antagonists (ARBs), β-blockers, and mineralocorticoid receptor antagonists (MRAs). While these therapies aim to improve the long-term prognosis of heart failure, they typically yield neutral or modest effects on symptoms. Recent years have witnessed the emergence of novel drugs aimed at improving health status (symptoms, function, and quality of life) and prognosis in heart failure patients. These innovative treatments encompass angiotensin receptor neprilysin inhibitors (ARNIs) [3], sodium-glucose co-transporter-2 inhibitors (SGLT-2i) [4–9], Ivabradine [10] and so on.

SGLT2i are a class of medications initially developed for managing type 2 diabetes mellitus (T2DM), which lower plasma glucose concentrations via increased urinary glucose excretion. Meanwhile, SGLT2i confer additional therapeutic benefits, such as weight reduction, decreased urinary albumin, and lowered blood pressure and uric acid levels [11, 12]. Meta-analysis [13, 14] have demonstrated that SGLT2i significantly reduce heart failure hospitalization, cardiovascular death, cardiovascular mortality, all-cause mortality and myocardial infarction in patients with or without diabetes mellitus, with a low incidence of adverse events.

In updated guidelines, SGLT2i (dapagliflozin, empagliflozin and sotagliflozin) are strongly recommended for reducing cardiovascular death and heart failure rehospitalization in patients with HFrEF (class I). However, recommendations for SGLT2is in HFpEF and HFmrEF are either absent or less robust (class II). While numerous clinical studies have explored SGLT2i in patients with CHF, with or without T2DM, only a limited few have investigated the early effects of SGLT-2i on HF disease-specific health status and cardiac function. Thus, the objective of this study was to identify and critically appraise clinical trials which used SGLT-2i as adjunct therapy to traditional treatment in CHF with or without T2DM. To this end we conducted a systematic review and meta-analysis of the identified trials, and herein discuss early effects of SGLT-2i on HF disease-specific health status and cardiac function, providing valuable scientific evidence to inform rational clinical application.

## Materials and methods

### Data sources and searches

An extensive search for clinical trials in PubMed, EMBASE, CENTRAL, Scopus, Web of Science, and two Chinese databases (CNKI and Wanfang) for RCTs (from inception through June 30,2023), Clinical Trials (http://www.clinicaltrials.gov) were also searched for the terms ‘SGLT2 inhibitor’, ‘sodium glucose cotransporter 2 inhibitor’, ‘*gliflozin’, ‘dapagliflozin’, ‘canagliflozin’, ‘empagliflozin’, ‘ertugliflozin’, ‘ipragliflozin’, ‘tofogliflozin’, ‘remogliflozin’, ‘sergliflozin’, ‘luseogliflozin’, ‘sotagliflozin’ was performed. The search strategy was adapted for each of the databases, and references of included studies were also reviewed.

### Study selection

**Fig. 1:**
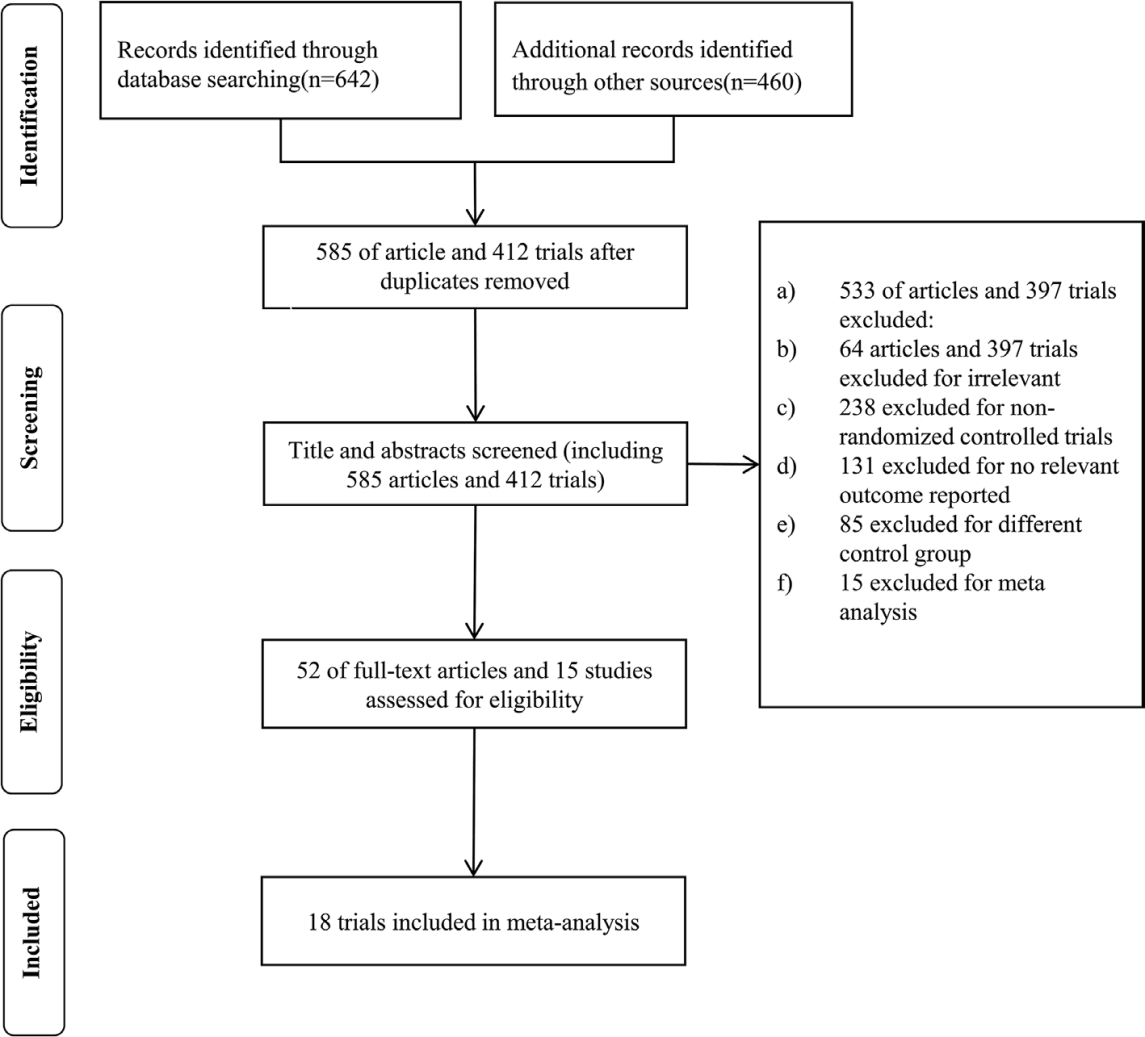
Flow chart of the literature search and study selection process

Two independent reviewers (Jiao Chen and Chunxia Jiang) conducted a thorough review of titles and abstracts to identify pertinent studies. Any discrepancies were resolved by discussion, with involvement of a third reviewer (Man Guo) when necessary. The study selection process is presented in Fig. 1. Included clinical trials compared SGLT-2i versus placebo in CHF patients (classified according to the European Society of Cardiology [1]) with or without T2DM (classified according to the American Diabetes Association [15]).

Eligible studies adhered to the following criteria: (a) randomized controlled trial (RCT) design, (b) participants aged 18 or older with CHF, with or without T2DM, (c) comparison of HF disease-specific health status and cardiac function between SGLT2i and placebo. Exclusion criteria encompassed duplicate publications, animal studies, reviews, conference abstracts, and meta-analysis.

Outcomes of interest included: (a) change in B-type natriuretic peptide (BNP) or N-terminal pro B-type natriuretic peptide (NT-proBNP), (b) proportion of patients achieved a meaningful reduction in BNP or NT-proBNP from baseline, (c) change in health status assessed via the Kansas City Cardiomyopathy Questionnaire (KCCQ) [16], a validated, self-administered, HF-specific instrument quantifying symptom frequency, symptom burden, symptom stability, physical limitations, social limitations, quality of life, and self-efficacy within a 2-week recall period based on 23 individual components, including KCCQ Total Symptom Score (KCCQ-TSS), KCCQ Overall Summary Score (KCCQ-OSS), and KCCQ Clinical Summary Score (KCCQ-CSS), (d) proportion of patients achieving a meaningful ≥ 5-point improvement in KCCQ-TSS, KCCQ-OSS, or KCCQ-CSS from baseline, (e) change in 6-minute walk distance from baseline, and (f) change in LVEF from baseline.

### Data extraction and quality assessment

All retrieved studies were managed using the reference management software EndNote 20.4.1. Two researchers (Jiao Chen and Chunxia Jiang) independently assessed the methodological quality of included clinical trials based on the Cochrane Collaboration Risk-of-Bias tool, recommended for quality assessment of the RCTs [17], and subsequently cross-checked these studies. Discrepancies were resolved through discussion or adjudicated by a third reviewer (Man Guo) when needed. Quality assessment followed the Cochrane Handbook’s criteria, which include sequence generation, allocation concealment, blinding, incomplete outcome data, and selective outcome reporting. Risk of bias was graded as unclear, high, or low.The judgement was not employed as a criterion for trial selection; certain items were used only for descriptive purpose.

### Data synthesis and analysis

Data analyses were conducted using RevMan 5.3 and Stata 17.0, tools developed by the Cochrane Collaboration. Weighted mean differences (WMDs) and risk ratios (RR) were calculated for continuous outcome variables (e.g., NT-proBNP) and dichotomous data (such as ≥ 20% decrease in NT-proBNP ≥ 5 points increase in KCCQ CSS), respectively. Heterogeneity was assessed using the Q test and I^2^ test, with I^2^ values of 25%, 50%, and 75% indicating low, medium, and high heterogeneity, respectively. The fixed effect model with the Mantel-Haenszel method was utilized when no statistical heterogeneity was present (I^2^ < 50% and p > 0.10). Conversely, the random-effects model was applied when heterogeneity was significant (I^2^ ≥ 50% or p < 0.10) [18]. Subgroup or sensitivity analyses were performed when necessary to explore sources of heterogeneity. Additionally, the random-effects model was used to address unexplained heterogeneity, and potential publication bias was evaluated through funnel plots.

## Results

### Study selection and characteristics

As illustrated in Fig. 1, a total of 642 articles and 460 trials were initially identified. Among these, a total of 18 RCTs met the inclusion criteria and were included in the meta-analysis. Out of these, 15 trials were reported in English, and 3 were reported in Chinese. The cumulative participant count across the 18 trials was 23,953, with 11,986 and 11,967 individuals allocated to the SGLT2i and placebo groups, respectively.

The baseline characteristics of the retrieved trials are presented in Table 1(additional information is summarized in Supplemental Table 1). The Follow-up duration ranged from 12 to 169 weeks. The mean age, body mass index (BMI), baseline LVEF and hemoglobin A1c (HbA1c), as analyzed from the available patient data, were 69.3 years, 29.2 kg/m², 43.8%, and 6.58%, respectively.

**Table 1:** Baseline characteristics of trials included in meta-analysis. CANA canagliflozin, DAPA dapagliflozin, EMPA empagliflozin, PBO placebo, left ventricular ejection LVEF, age and LVEF were reported in Mean, * for median(Q1,Q3), NR, not reported

T2D,n (%)	166(63.1)	1466(58.9)	1856(49.8)	0(0)	2806(44.8)	25(13.1)	125(27.9)	634(52.3)	84(80)
LVEF, %	26.4(8.1)	54.3(8.8)	27.4(6.1)	36.3(8.1)	54.2(8.8)	29.5(8.0)	NR	43.7(16.2)	32.5(9.8)
Male, n (%)	193(73.4)	3312(55.3)	2837(76.1)	54(64.3)	3516(56.1)	162(85.3)	251(55.2)	11(63.1)	77(73.3)
Age,years	61.3(11.5)	71.9(9.4)	66.8(11.0)	62(12.1)	71.7(9.6)	64(8.5)	63.4(13.3)	66.2(12.9)	68.7(11.1)
Interventions	DAPA 10 mg, PBO	EMPA 10 mg, PBO	EMPA 10 mg, PBO	EMPA 10 mg, PBO	DAPA 10 mg, PBO	EMPA 10 mg, PBO	CANA 100 mg, PBO	EMPA 10 mg, PBO	EMPA 10 mg, PBO
Participants,n	263	4988	3730	84	6263	190	448	65	105
Follow-up,weeks	52	52	148	26	169	12	12	12	36
Condition	HFrEF	HFpEF	HFrEF	HFrEF	HFpEF	HFrEF	HF	HF	HFrEF
Country	United States	United States	United States	United States	United States	Denmark	United States	United States	United Kingdom
Completion Date	2019/6/28	2021/4/26	2020/5/28	2020/2/13	2022/3/27	2020/1/17	2021/11/09	2020/03/10	2020/03/18
NCT ID	NCT02653482	NCT03057951	NCT03057977	NCT03485222	NCT03619213	NCT03198585	NCT04252287	NCT03030222	NCT03485092
Study	DEFINE-HF [19, 20]	EMPEROR-Preserved [21–23]	EMPEROR-Reduced [8, 24, 25]	EMPA-TROPIS M [26, 27]	DELIVER [28, 29]	Empire HF [30, 31]	CHIEF-HF [32, 33]	EMBRACE-HF [34]	SUGAR-DM-HF [35]
T2D,n (%)	1983(41.8)	181(55.9)	NR	NR	NR	NR	73(49.7)	50(100)	29(25.9)
LVEF, %	31.1(6.8)	60(54, 65)*	NR	NR	NR	NR	28.3(7.2)	47.3(9.1)	45.5(3.3)
Male, n (%)	3635(76.6)	140(43.2)	232(74.4)	179(56.8)	233(74.4)	320(63.5)	112(76.2)	28(56)	84(75)
Age,years	66.3(10.9)	70(63, 77)*	69.0(10.2)	73.5(8.8)	67.8(10.4)	71.8(9.4)	61.9(11.6)	66.1(7.1)	69(57,78)*
Interventions	DAPA 10 mg, PBO	DAPA 10 mg, PBO	EMPA 10 mg, PBO	EMPA 10 mg, PBO	DAPA 10 mg, PBO	DAPA 10 mg, PBO	DAPA 10 mg, PBO	DAPA 10 mg,CA NA 100 mg, PBO	EMPA 10 mg, PBO
Participants,n	4744	324	312	313	313	504	147	50	112
Follow-up,weeks	104	12	12	12	16	16	52	26	83
Condition	HFrEF	HFpEF	HFrEF	HFpEF	HFrEF	HFpEF	HFrEF	HFrEF	HFrEF
Country	United States	United States	United States	United States	United States	United States	China	China	China
Completion Date	2019/7/17	2021/8/13	2019/10/07	2019/10/09	2020/03/07	2020/07/09	2020/10/31	2020/12/31	2021/04/30
NCT ID	NCT03036124	NCT03030235	NCT03448419	NCT03448406	NCT03877237	NCT03877224	NR	NR	NR
Study	DAPA-HF [7, 36, 37]	DAPA-Preserved [38, 39]	EMPERIAL-Reduced [40, 41]	EMPERIAL-Preserved [40, 41]	DETERMINE-reduced [42]	DETERMINE-Preserved [43]	Zheng et al [44]	Li et al [45]	Wu et al [46]

### Risk of bias and publication bias

The risk of bias assessment for all included trials is summarized in Supplementary Figure S1. Most studies demonstrated adequate random sequence generation and allocation concealment, resulting in a low risk of bias. No significant evidence of publication bias was observed.

### Results

#### Effects on cardiac function

##### Change in NT-proBNP

**Fig. 2:**
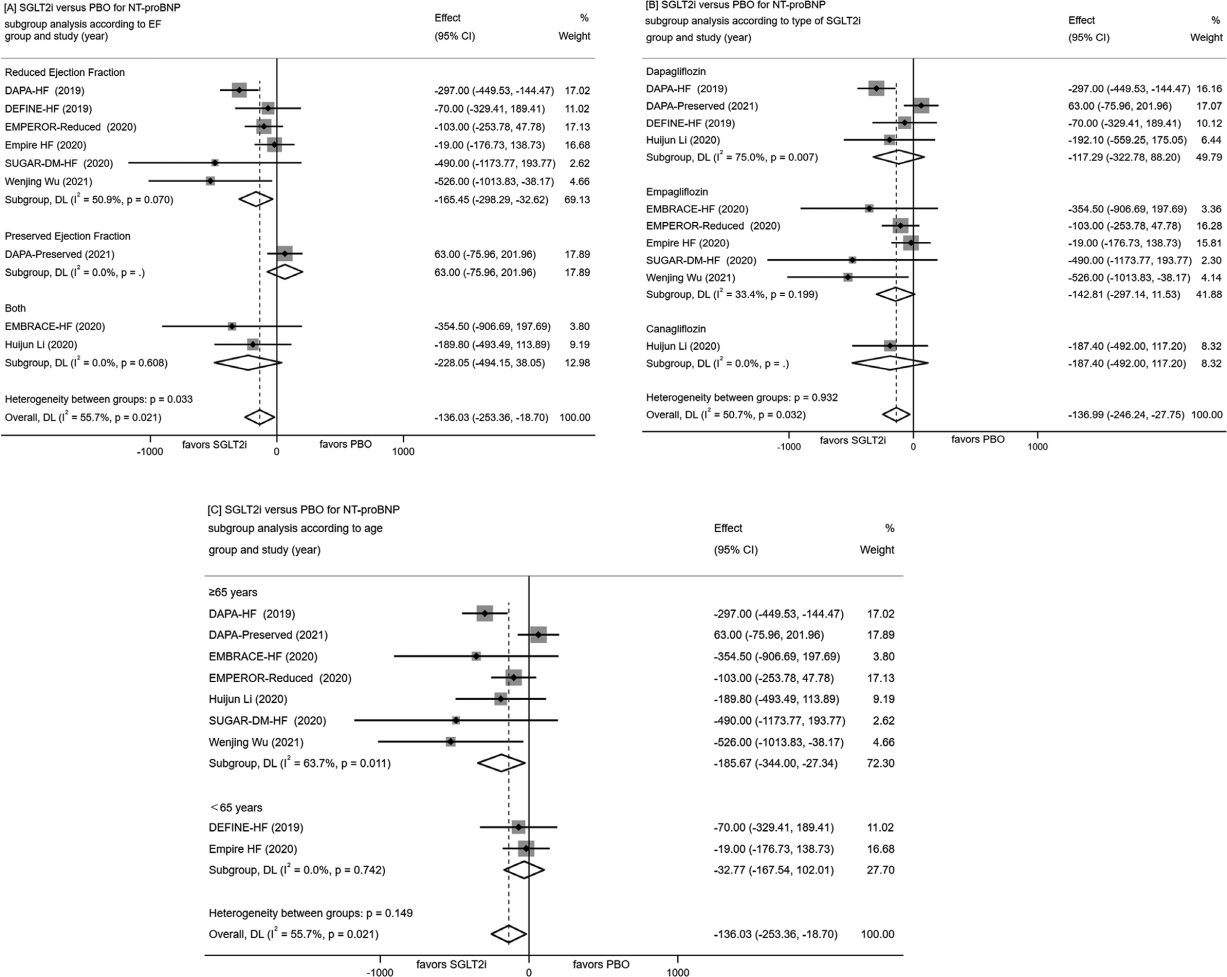

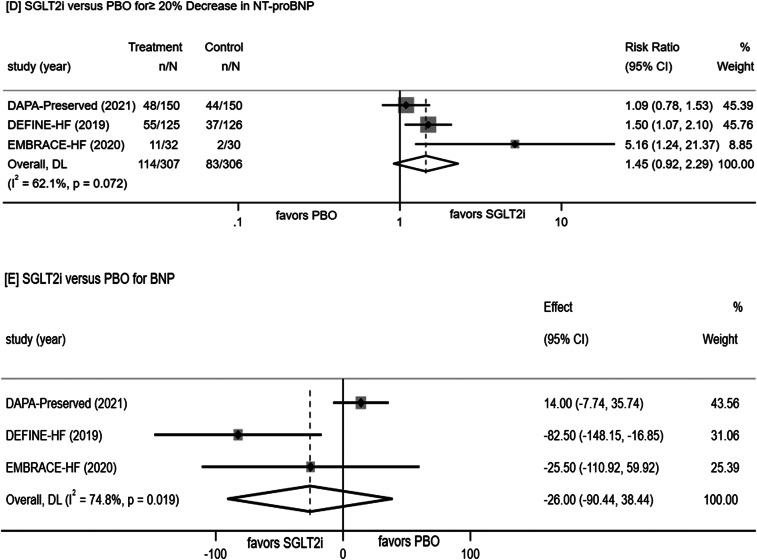
Forest plot for meta-analysis and subgroup analysis according to EF(**A**), type of SGLT2i(**B**) and age(**C**), comparing the effects of SGLT2i with PBO in NT-proBNP, ≥ 20% Decrease in NT-proBNP(**D**) and BNP(**E**). SGLT2i, SGLT-2 inhibitor, PBO, placebo, Effect stands for WMD = Weighted Mean Difference, CI confidence interval, RR = risk ratio

A meta-analysis of nine RCTs revealed that the SGLT-2 inhibitors group exhibited a statistically significant reduction in NT-proBNP by 136.03 pg/mL [− 253.36, − 18.70] compared to the placebo control (P = 0.021). Further subanalysis of the results according to ejection fraction(EF), type of SGLT2i and age were also conducted to explore their potential effects on NT-proBNP in SGLT2i or PBO group. It seems that a greater improvement in reduced and preserved ejection fraction both, canagliflozin and ≥ 65 years patients in SGLT2i group compared with PBOas shown in Fig. 2A-C.

The occurrence of ≥ 20% decrease in NT-proBNP was 37.1% (114 out of 307) in the SGLT-2 inhibitors group and 27.1% (83 out of 306) in the placebo control, with a risk ratio (RR) of 1.45 (95% CI [0.92, 2.29], P = 0.072; Fig. 2D).

##### Change in BNP

Conversely, the summary of results from three RCTs investigating the effects of SGLT-2 inhibitors versus placebo control on BNP demonstrated high heterogeneity (P = 0.02, I² = 75%). The random-effects model analysis did not reveal a statistically significant difference in the effects on BNP between SGLT-2 inhibitors and placebo controls, as illustrated in Fig. 2E.

##### Change in LVEF

**Fig. 3:**
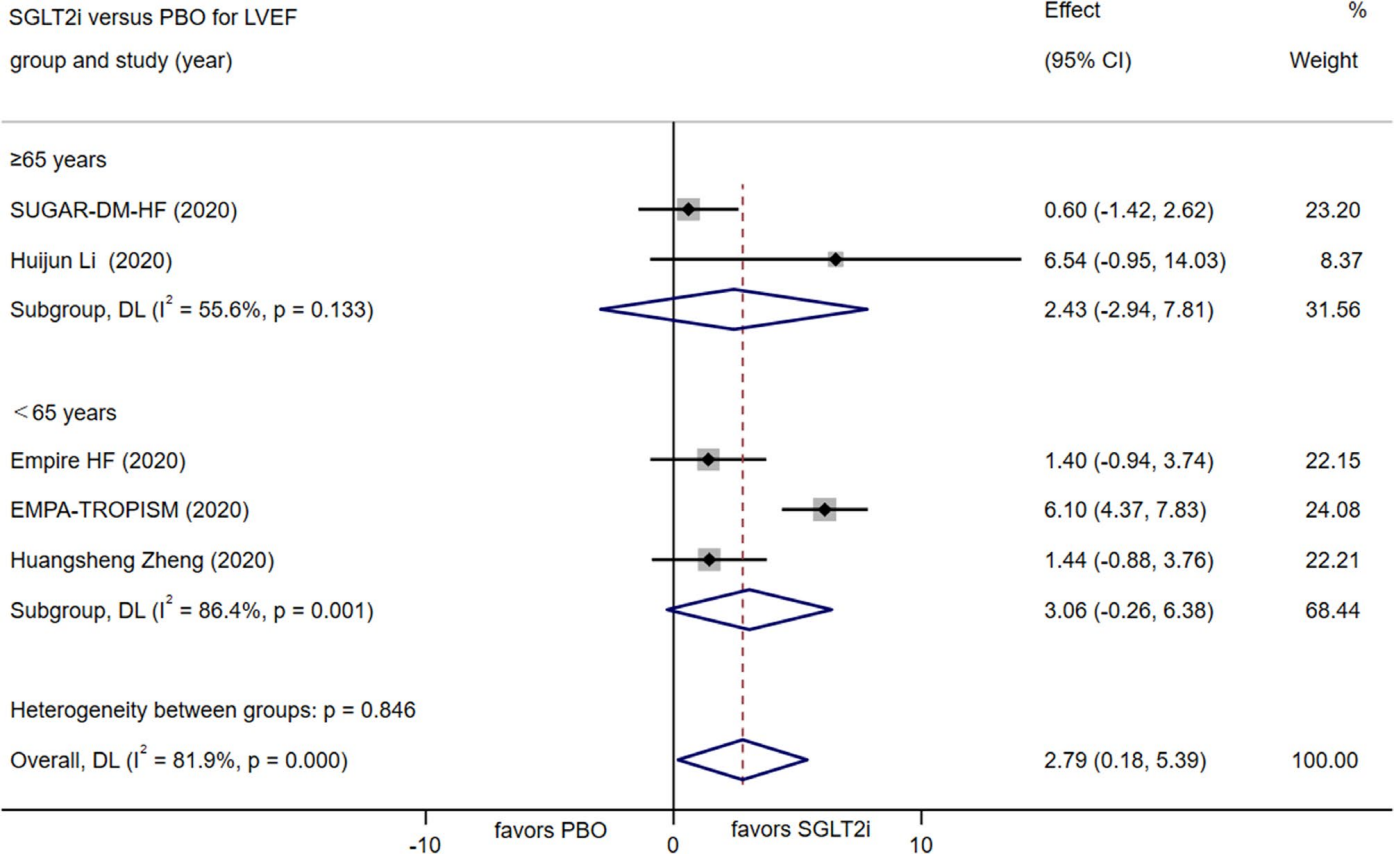
Forest plot for meta-analysis and subgroup analysis according to age, comparing the effects of SGLT2i with PBO on LVEF. SGLT2i, SGLT-2 inhibitor, PBO, placebo, Effect stands for WMD = Weighted Mean Difference, CI confidence interval

Five RCTs investigated the effects of SGLT-2 inhibitors versus placebo control on LVEF. The use of SGLT-2 inhibitors led to a noteworthy improvement in LVEF by 2.79% (95% CI [0.18, 5.39], P = 0.036). A further subgroup analysis according to age were conducted considering the high heterogeneity (I2 = 81.9%). The random-effects model analysis did not reveal a statistically significant difference in the effects on subgroup analysis between <65 years and ≥ 65 year, as illustrated in Fig. 3.

#### Effects on health status

##### Change in KCCQ-CS

**Fig. 4:**
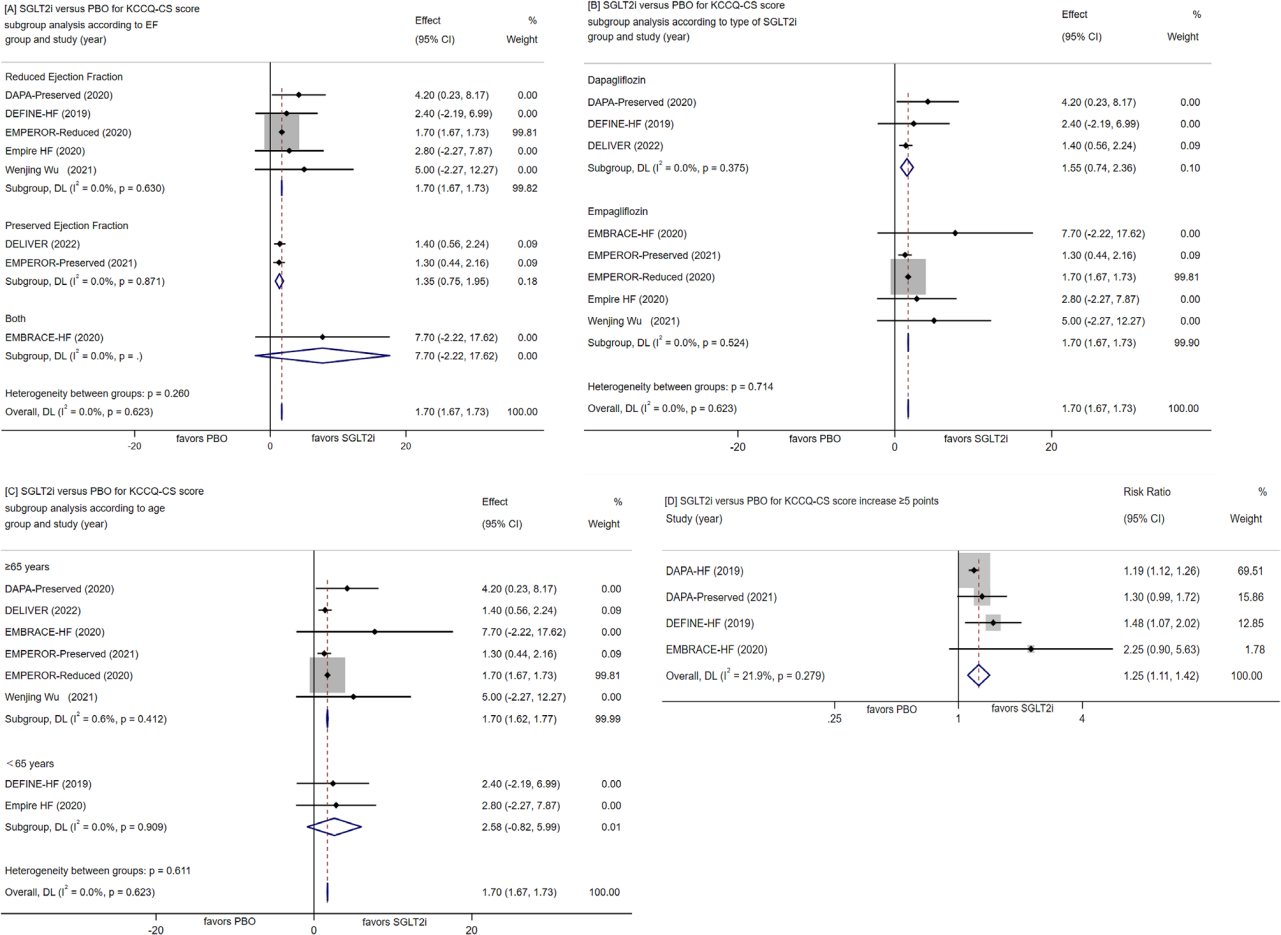
Forest plot for meta-analysis and subgroup analysis according to EF(**A**), type of SGLT2i(**B**) and age(**C**), comparing the effects of SGLT2i with PBO in KCCQ-CS score KCCQ-CS score increase ≥ 5 points(**D**). SGLT2i, SGLT-2 inhibitor, PBO, placebo, Effect stands for WMD = Weighted Mean Difference, CI confidence interval, RR = risk ratio

An assessment of eight RCTs indicated that the use of SGLT-2 inhibitors led to a noteworthy improvement in KCCQ-CS score (WMD = 1.7, 95% CI [1.67, 1.73], P < 0.00001; Fig. 4A-C). Notably, the most substantial score elevation was observed with Empagliflozin 10 mg once daily, resulting in an increase of 8.0 over a 6-month follow-up period [46]. Furthermore, when compared to placebo, the SGLT-2 inhibitors group exhibited an RR of 1.25 (95% CI [1.11, 1.42], P < 0.00001; Fig. 4D) for KCCQ-CS score increase ≥ 5 points, with 1404 cases (53.2%) in the SGLT-2 inhibitors group and 1160 cases (43.3%) in the placebo group. We also make a further subanalysis of the results according to EF, type of SGLT2i in SGLT2i or PBO group. It seems that a greater improvement in reduced and preserved ejection fraction both, empagliflozin and <65 years patients in SGLT2i group compared with PBO.

##### Change in KCCQ-OS score

**Fig. 5:**
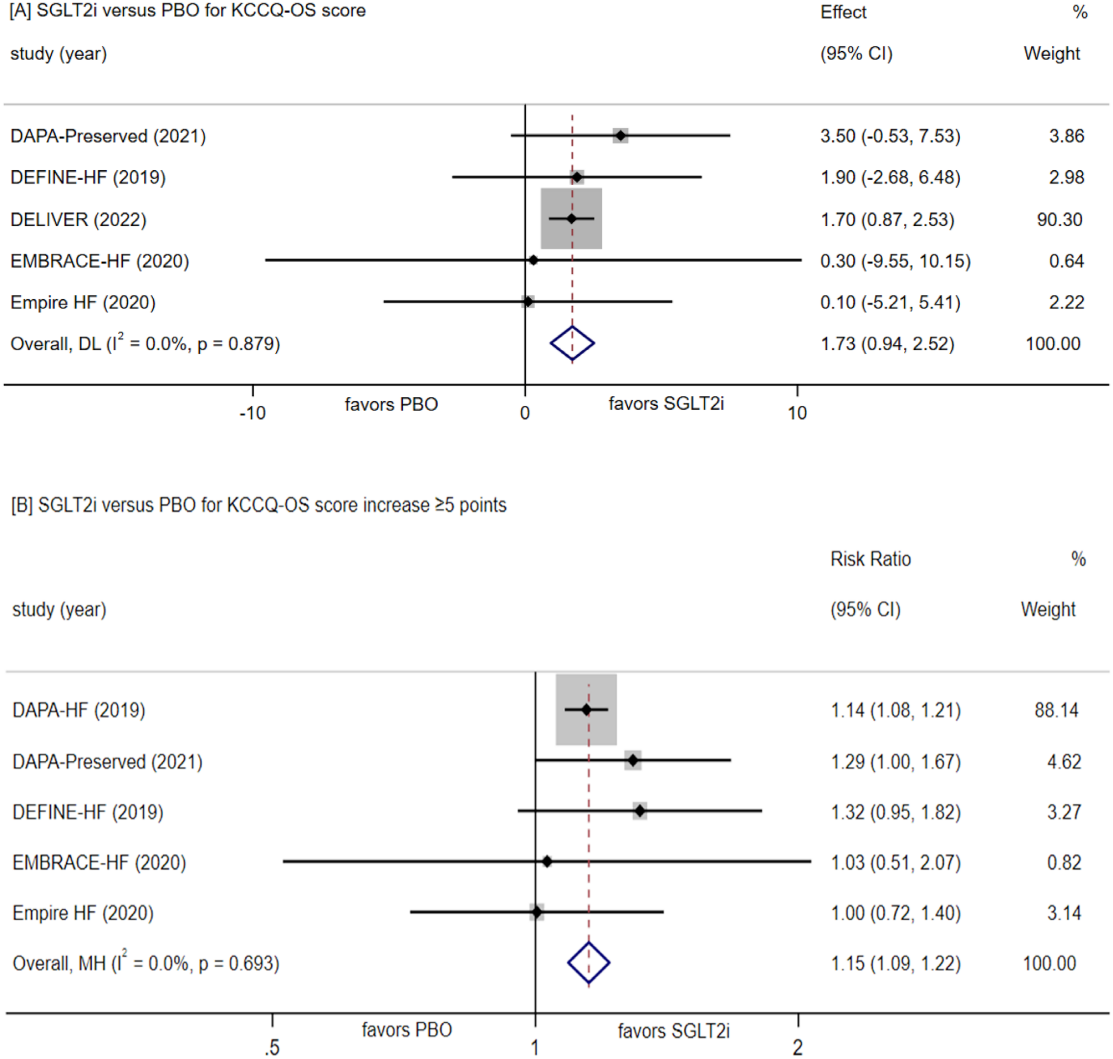
Forest plot for meta-analysis comparing the effects of SGLT2i with PBO in KCCQ-OS score(**A**), KCCQ-OS score increase ≥ 5 points(**B**). SGLT2i, SGLT-2 inhibitor, PBO, placebo, Effect stands for WMD = Weighted Mean Difference, CI confidence interval, RR = risk ratio

Throughout the follow-up period, the SGLT-2 inhibitors group displayed a mean KCCQ-OS score increase ranging from 0.94 to 2.52 (p < 0.0001 versus placebo; Fig. 5A). A total of five RCTs reported events of KCCQ-OS score increase ≥ 5 points. In this context, the SGLT-2 inhibitors group exhibited an RR of 1.15 (95% CI [1.09, 1.22], P < 0.00001; Fig. 5B) compared to placebo, with 1445 (52.0%) cases in SGLT-2 inhibitors and 1253 cases (45.2%) in placebo.

##### Change in KCCQ-TS score

**Fig. 6:**
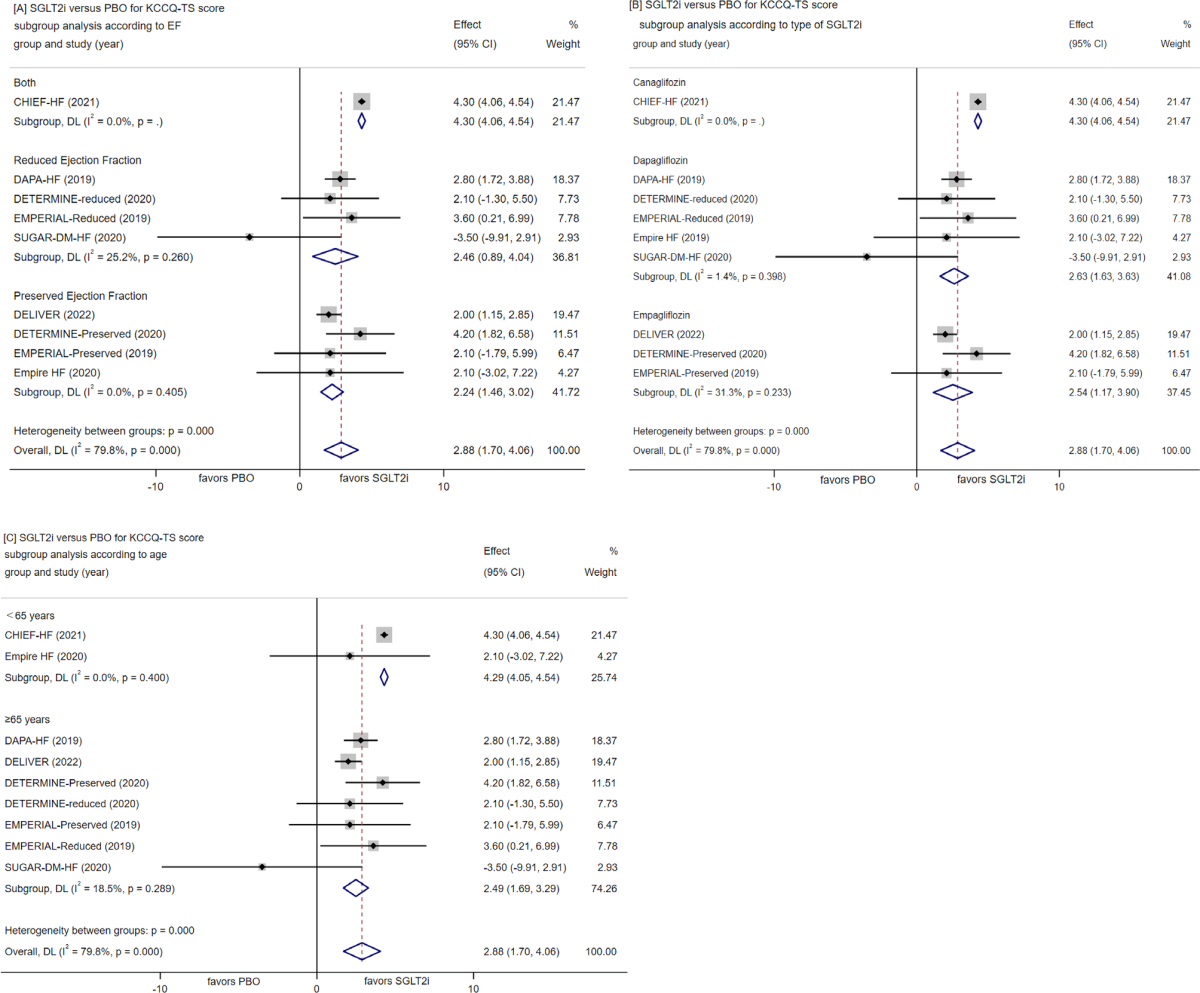
Forest plot for meta-analysis and subgroup analysis according to EF(**A**), type of SGLT2i(**B**) and age(**C**), comparing the effects of SGLT2i with PBO in KCCQ-TS score. SGLT2i, SGLT-2 inhibitor, PBO, placebo, Effect stands for WMD = Weighted Mean Difference, CI confidence interval

Nine RCTs evaluated the effects of SGLT-2 inhibitors versus placebo on KCCQ-TS score. I Across these trials, the use of SGLT-2 inhibitors resulted in a significant improvement in KCCQ-TS score (WMD = 2.88, 95% CI [1.7, 4.06], P < 0.00001; Fig. 6A-C). Considering the high heterogeneity (I^2^ = 79.8%, p = 0.0001), a meta-regression analysis was conducted to explore potential causes of heterogeneity. This analysis examined four factors that may affect heterogeneity, including EF, the type of SGLT2 inhibitors, age, and follow-up time. It was found that EF (P = 0.029), type of SGLT2 inhibitors (P = 0.029) and age (P = 0.007) might contribute to study heterogeneity. Subgroup analysis was subsequently performed based on EF(reduced ejection fraction, preserved ejection fraction and both), age (≤ 65 years, > 65 years) and SGLT2 inhibitor type (canagliflozin, dapagliflozin, empagliflozin), revealing significant changes in WMD values associated with these factors.

##### Change in 6-minute walk distance

**Fig. 7:**
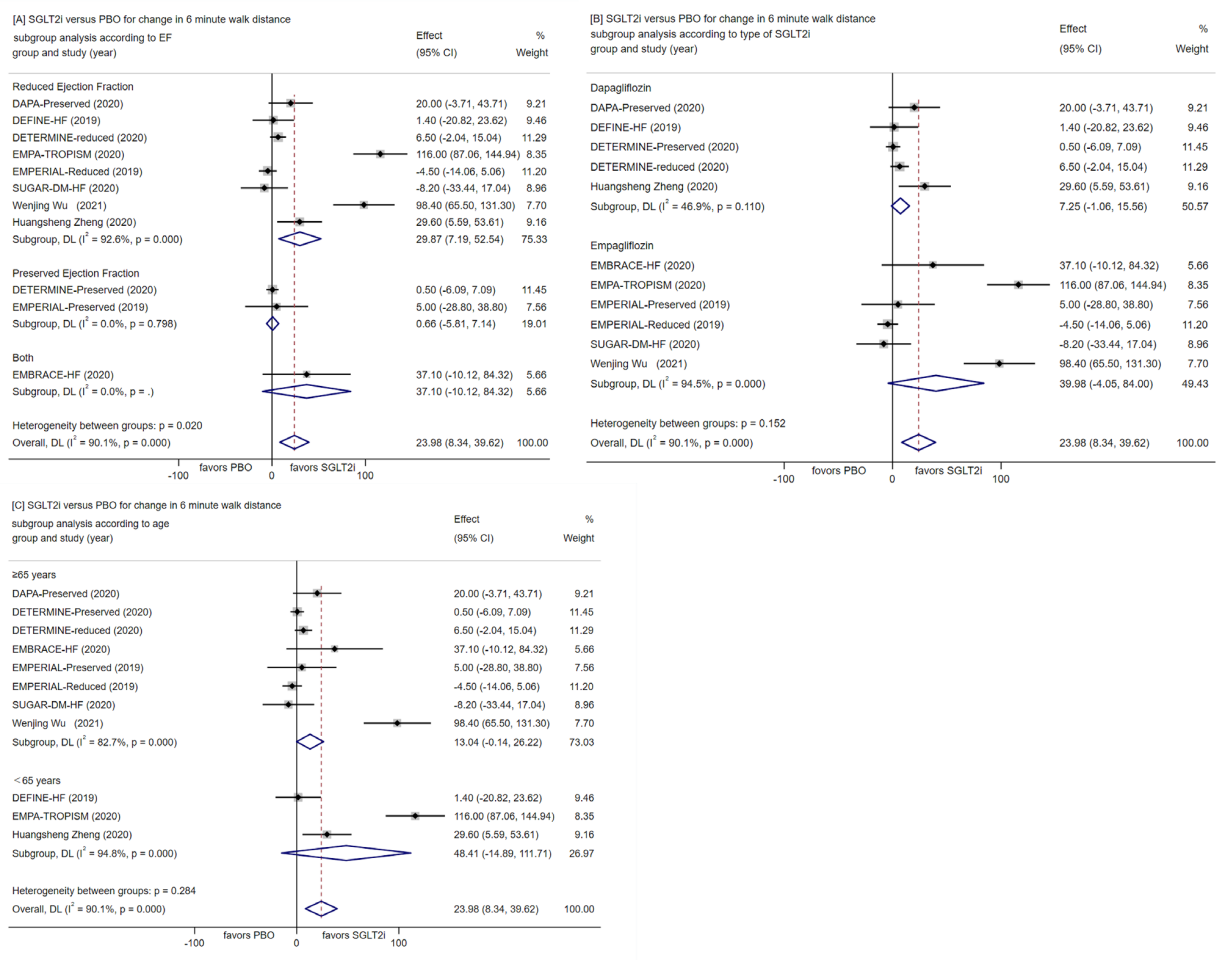
Forest plot for meta-analysis and subgroup analysis according to EF(**A**), type of SGLT2i(**B**) and age(**C**), comparing the effects of SGLT2i with PBO for change in 6 min walk distance. SGLT2i, SGLT-2 inhibitor, PBO, placebo, Effect stands for WMD = Weighted Mean Difference, CI confidence interval

Overall, SGLT2 inhibitors demonstrated an increase of 23.98 m in the 6-minute walk distance (heterogeneity: I² = 90%, P < 0.00001; 95% CI [8.39, 39.62], p = 0.003) compared to control/placebo from baseline (Fig. 7A-C). Notably, empagliflozin 10 mg once daily exhibited an increase of 149.7 m over 83 weeks [46]. Meta-regression analysis sought to identify potential sources of heterogeneity, considering baseline EF, type of SGLT2 inhibitors, follow-up time (≤ 26 weeks, 26–52 weeks, > 52 weeks) participant age (≤ 65 years, > 65 years), and whether patients were complicated with T2D. Findings indicated that WMD values did not significantly change with these factors. Funnel plots indicated noticeable heterogeneity in trials of EMPA-TROPISM and Wenjing Wu et al.

In order to explore potential facts that might influence SGLT2i’ effects on cardiac function and health status, we sum up the outcomes according to gender, with or without T2D. In EMPEROR-Preserved [23], empagliflozin further improved KCCQ-CSS compared with placebo, adjusted mean change from baseline by 1.67(95% CI,0.42 to 2.91) in patients with diabetes and 0.99(95% CI, -0.22 to 2.20) without diabetes. In EMBRACE-HF [38], empagliflozin further improved KCCQ-CSS with empagliflozin compared with placebo, adjusted mean change from baseline by 7.3(95% CI, 2.3 to 12.3) in patients with diabetes and 2.9(95% CI, -2.1 to 7.8) without diabetes, all P values for interaction are nonsignificant. A greater proportion of patients treated with dapagliflozin had a clinically meaningful improvement of ≥ 5 points in KCCQ-OS or at least a 20% reduction in NT-proBNP, as compared with placebo (61.5% vs. 50.4%, adjusted OR 1.8; 95% CI, 1.03 to 3.06, P = 0.039), the results were consistent within subgroups of patients with and without T2D [20]. The effects of canagliflozin on the change in the KCCQ TSS at 12 weeks were consistent in patients with T2DM (6.5; 95% CI, − 0.2 to 13.2) and participants without T2DM (3.6; 95% CI, − 0.5 to 7.8) (P value for interaction = 0.90) [32]. Meanwhile, EMBRACE-HF and DEFINE-HF showed that there was no difference between empagliflozin/dapagliflozin and placebo in change for KCCQ-CSS or improvement of ≥ 5 points in KCCQ-OS or at least a 20% reduction in NT-proBNP in male or female.

## Disscusion

Clinical trial data and further meta-analysis have proven the efficiency and safety of SGLT2 inhibitors as monotherapy or in combination with other therapies (metformin, DPP-4 inhibitors, GLP-1 agonists, insulin) for managing T2DM [47–50]. These inhibitors have demonstrated the ability to reduce the risk of cardiovascular and all-cause mortality or worsening HF in patients with CHF patients [51]. Furthermore, recent trials investigating SGLT2 inhibitors in acute heart failure with or without diabetes (NCT03200860, NCT04157751, NCT03521934, NCT04298229) have yielded promising results. A meta analysis [52] involving 1831 subject globally demonstrated that initiation of SGLT2 inhibitors in patients hospitalized for AHF during hospitalization or early post-discharge (within 3 days) reduces the risk of rehospitalization for heart failure and improves patient-reported outcomes without additional adverse effects.

Improving symptom burden is a critical goal for HF management. Yet, the quantifiable influence of SGLT2 inhibitors on symptom burden, physical function, and quality of life in HF patients with or without T2DM remains uncertain. Although only dapagliflozin and empagliflozin have received HF indications thus far, multiple studies offer significant supporting evidence regarding the beneficial effects of SGLT2 inhibitors when added to standard HF treatment, manifesting as early as two weeks following therapy initiation.

The present meta-analysis includes the most recent published large RCTs (EMPEROR-Preserved, EMPEROR-Reduced, DELIVER, DAPA-HF) thus providing the most contemporary assessment of the total available evidence for SGLT-2 inhibitor therapy and cardiac function or health status outcomes in CHF patients with or without T2D. The findings of 18 RCTs involving 23,953 patients show that treatment with SGLT-2 inhibitors reduced NT-proBNP by 136.03pg/ml and improve LVEF by 2.79% in the overall population, while no statistically significant difference was observed for the effects on BNP. A further subgroup analyses indicating significant difference in the reduced NT-proBNP in patients with HFrEF or HFpEF.

In addition to the observed benefits on LVEF, we summed up the SGLT2i’ effects on many other cardiac morphological index based on the available data. SUGAR-DM-HF, Empire HF and EMPA-TROPISM found that treatment of patients with HFrEF with empagliflozin led to a significant reduction in LV end-systolic volume index (LVESVi, between-group difference − 4.9ml/m2) ,LV end-diastolic volume index(LVEDVi ,between-group difference − 6.4ml/m2), LV end-systolic volume(LVESV, between-group difference − 13.6ml) and LV end-diastolic volume(LVEDV, between-group difference − 15.6ml) compared with placebo, larger sample sizes, higher quality data, and studies on cardiac morphological index are needed for future clinical research.

Patients treated with SGLT2i generally experienced somewhat higher KCCQ scores (especially the KCCQ-TS score) and 6-min walking distances compared with placebo. Subgroup analyses further suggest that baseline EF and age significantly affect KCCQ-TS score, baseline EF also significantly affect 6-minute walk distance.

Several potential mechanisms may explain the clinical benefits of SGLT2 inhibitors. First, SGLT2 inhibitors’ inhibition of glucose and sodium reabsorption in the proximal tubule, leading to a modest osmotic diuretic effect, thus have been shown to lower pulmonary artery pressure, which aids decongestion and can translate to improvements in both **s**ymptoms and exercise function [34, 53]. Second, SGLT2 inhibitors may increase myocardial energy production, alter substrate utilization and cellular signaling though increased lipolysis in adipose tissue with subsequent generation of ketone bodies [54]; reduc the leakage of Ca^2+^ from sarcoplasmic reticulum (SR) thereby enhancing Ca^2+^ transient amplitude in cardiomyocytes and improving diastolic function [55]; improve systemic endothelial function [56]; reduc oxidative stress and inflammation in HFpEF cardiomyocytes, coupled with improved endothelial vasorelaxation, ultimately enhancing ventricular relaxation [56, 57].

### Study limitations

Several limitations warrant consideration in this study. First, although we attempted to explore potential sources of heterogeneity and conduct subgroup analyses based on EF, SGLT2 inhibitor type, follow-up duration, participant age, and other factors, while we failed to explain all possible heterogeneities due to inherent differences in characteristics, definitions of the included studies. Second, most study participants were from Western countries, which limited the applicability of the results to other ethnic groups such as Asians and Africans. Third, most of the included studies had follow-up periods of less than 52 weeks. Moreover, not all RCTs have published the subgroup data for all outcomes and therefore, subgroup analysis according to gender, patients with diabetes or without diabetes, the presence of previous cardiovascular disease or not was failed.

## Conclusions

These findings suggest that the SGLT2 inhibitors treatment offers an optimal strategy for improving NT-proBNP and health status (assessed by Kansas City Cardiomyopathy Questionnaire and 6-minute walk distance) in CHF patients with or without T2DM. Which will provide valuable clinical insights to guide treatment decisions for healthcare professionals.

### Electronic supplementary material

Below is the link to the electronic supplementary material.

Supplementary Material 1

Supplementary Material 2

## Data Availability

All data were extracted from publicly available sources and are included in this published article and its Additional file.
