# Supplementary figures and images for: Effects of SGLT2 inhibitors on cardiac function and health status in chronic heart failure: a systematic review and meta-analysis
Source: Cardiovasc Diabetol. 2024 Jan 3;23:2. doi: 10.1186/s12933-023-02042-9 (PMC10765651; doi:10.1186/s12933-023-02042-9)

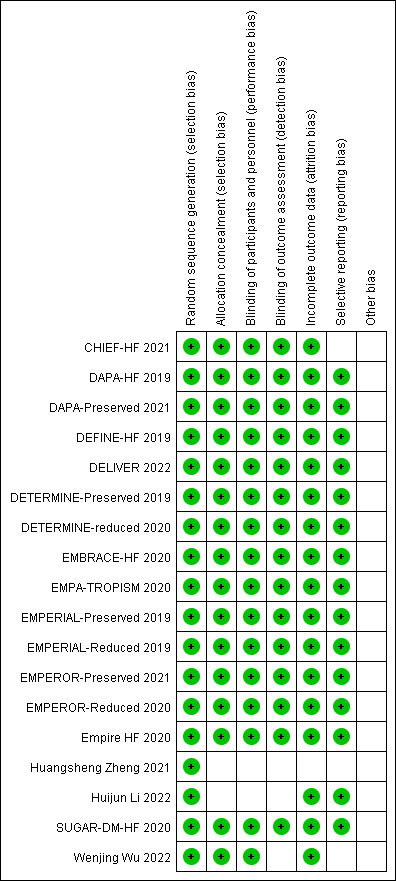

Supplement: Supplementary file 1 — Supplementary Material 1 [file 12933_2023_2042_MOESM1_ESM.png]
